# Foreign Body Pneumonia

**Published:** 1916-03

**Authors:** 


**Foreign Body Pneumonia.** A. Caillé, N. Y., Arch of Paed., Dec. 1915, reports four cases in young children. Spasmodic cough, expectoration of pus in acute lesion, without amphoric respiration or other indication of cavity, should lead to X-ray examination.

